# Treg-Resistant Cytotoxic CD4^+^ T Cells Dictate T Helper Cells in Their Vicinity: TH17 Skewing and Modulation of Proliferation

**DOI:** 10.3390/ijms22115660

**Published:** 2021-05-26

**Authors:** Cindy Hoeks, Marjan Vanheusden, Liesbet M. Peeters, Piet Stinissen, Bieke Broux, Niels Hellings

**Affiliations:** 1 Neuro Immune Connections & Repair Lab, Department of Immunology and Infection, Biomedical Research Institute, Hasselt University, Martelarenlaan 42, 3500 Hasselt, Belgium; cindy.hoeks@uhasselt.be (C.H.); Marjan.Vanheusden@gymna-uniphy.com (M.V.); liesbet.peeters@uhasselt.be (L.M.P.); piet.stinissen@uhasselt.be (P.S.); bieke.broux@uhasselt.be (B.B.); 2 Universitair MS Centrum (UMSC), Martelarenlaan 42, 3500 Hasselt, Belgium

**Keywords:** cytotoxic CD4 T cells, CD4 CTL, regulatory CD4 T cells, suppression, proliferation, autoimmunity

## Abstract

Cytotoxic CD4^+^ T cells (CD4 CTL) are terminally differentiated T helper cells that contribute to autoimmune diseases, such as multiple sclerosis. We developed a novel triple co-culture transwell assay to study mutual interactions between CD4 CTL, conventional TH cells, and regulatory T cells (Tregs) simultaneously. We show that, while CD4 CTL are resistant to suppression by Tregs in vitro, the conditioned medium of CD4 CTL accentuates the suppressive phenotype of Tregs by upregulating IL-10, Granzyme B, CTLA-4, and PD-1. We demonstrate that CD4 CTL conditioned medium skews memory TH cells to a TH17 phenotype, suggesting that the CD4 CTL induce bystander polarization. In our triple co-culture assay, the CD4 CTL secretome promotes the proliferation of TH cells, even in the presence of Tregs. However, when cell−cell contact is established between CD4 CTL and TH cells, the proliferation of TH cells is no longer increased and Treg-mediated suppression is restored. Taken together, our results suggest that when TH cells acquire cytotoxic properties, these Treg-resistant CD4 CTL affect the proliferation and phenotype of conventional TH cells in their vicinity. By creating such a pro-inflammatory microenvironment, CD4 CTL may favor their own persistence and expansion, and that of other potentially pathogenic TH cells, thereby contributing to pathogenic responses in autoimmune disorders.

## 1. Introduction

Cytotoxic CD4^+^ T cells (CD4 CTL) arise during the chronic activation of the immune system. The hallmark of CD4 CTL is the loss of CD28 expression, induced by repeated antigenic stimulation, caused, for instance, by latent viruses such as cytomegalovirus. This coincides with restricted TCR diversity and oligoclonality [1,2,3,4,5,6]. At a functional level, CD4 CTL are co-stimulation independent, resistant to apoptosis, and less susceptible to suppression by regulatory T cells (Tregs) [4,6,7,8,9]. They are suspected of contributing to many inflammatory diseases, due to their cytotoxic capabilities via the expression of natural killer cell receptors and the production of perforin and granzymes, their ability to infiltrate target tissues, such as the central nervous system, using the fractalkine receptor CX3CR1, and their autoreactive nature [9,10,11,12,13]. To illustrate this, we previously found that expansion of this T cell subset correlates with disability in experimental autoimmune encephalomyelitis, the animal model of multiple sclerosis, a demyelinating autoimmune disease of the central nervous system [14]. In addition, we found that expansion of CD4 CTL correlates with worse prognosis in multiple sclerosis patients [15].

One of the most striking features of CD4 CTL is their resistance to Treg-mediated suppression in vitro [6]. It is, however, still unclear which mechanisms CD4 CTL utilize to evade Treg suppression. Candidate pathways can be roughly divided into two separate modes of actions, with the first mode being the active induction of Treg dysfunction by CD4 CTL, and the second one being an intrinsic decreased susceptibility of CD4 CTL to Treg-mediated suppression. Regarding the induction of Treg dysfunction, several molecular interactions can be involved [16,17,18]. For instance, the expression by Tregs of co-inhibitory molecules (CTLA4, PD-1, LAG3) [19,20,21], immunosuppressive cytokines and their enhancers (TGFβ/GARP, IL-10) [22,23,24,25], apoptosis-related molecules (granzyme, FasL) [26,27], and molecules implicated in metabolic disruption (CD39, A_2_AR receptor) [28,29,30] might be affected as a result of interaction with CD4 CTL. Tregs could also upregulate proinflammatory cytokines, such as IFN-γ in response to CD4 CTL, which render them less suppressive [31,32]. These phenotypic changes in Tregs could be induced by the proinflammatory and cytotoxic microenvironment created by CD4 CTL, where increased levels of IFN-γ, IL-1β, and GrB are found [16,33,34,35]. Furthermore, CD4 CTL might express molecules that favor the development of T helper (TH) 17 effector cells over Tregs, such as IL-6, IL-22, and GM-CSF [34,36,37,38]. Second, the reduced susceptibility of CD4 CTL to Treg suppression can be induced by several molecular interactions. The reduced expression of IL-10R might directly render them less sensitive to IL-10-mediated suppression [16,17], while increased expression of the transcription factor HOPX might positively affect CD4 CTL survival [39]. Indirectly, reduced expression of costimulatory molecules such as CD28 and GITR on CD4 CTL could inhibit Tregs via increased exposure of adjacent Tregs to shared ligands CD80 and GITRL expressed on antigen-presenting cells [40,41].

To elucidate which of these pathways are involved in the evasion of Treg-mediated suppression by CD4 CTL, we investigated how CD4 CTL interact with Tregs and CD28^+^ TH cells, which are further referred to as conventional TH cells. We characterized the phenotype of activated CD4 CTL, as well as the phenotype of Tregs and conventional TH when exposed to the conditioned medium of CD4 CTL. Additionally, we utilized in vitro co-culture systems to study the interaction of these three cell types. Building further on the well-established conventional suppression assays in which proliferation of one responder T cell subset is analysed in the absence or presence of Tregs, we developed a triple co-culture assay which allowed us to study CD4 CTL, conventional TH, and Tregs simultaneously. Finally, addition of the transwell set-up to this triple co-culture assay made it possible to study the importance of cell−cell contact in the interaction of CD4 CTL with other CD4^+^ T cells. This unique set-up enabled us to provide more insight into how the peculiar CD4 CTL subset influences their microenvironment.

## 2. Results

### 2.1. CD4 CTL Resist Treg-Mediated Suppression Independent of Their Expression of IL-10R and GITR

**Figure 1:**
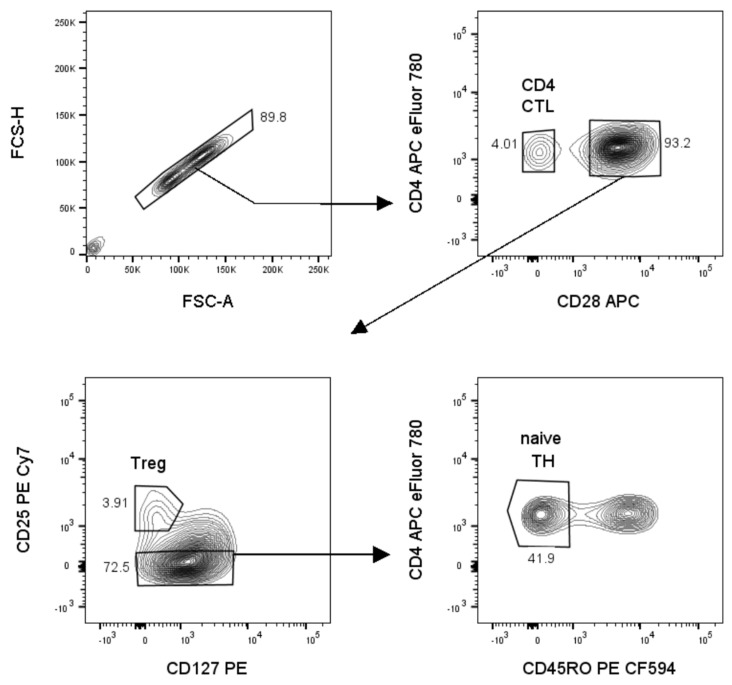
Sort strategy to sort three populations simultaneously: naïve TH, cytotoxic CD4 T cells (CD4 CTL) and regulatory T cells (Tregs). Prior to sorting, the total CD4^+^ T cell fraction is isolated from fresh PBMCs using magnetic beads. On the CD4^+^ T cell fraction, single cells are gated, using the area and height of the forward scatter (FSC-A, FSC-H). Next, CD4^+^CD28^+^ and CD4^+^CD28^–^ (CD4 CTL) cells are gated. Within the CD4^+^CD28^+^ gate, Tregs are gated as CD25^hi^CD127^–^. Finally, naïve TH are gated within the CD127^+^ population as CD45RO^–^.

**Figure 2:**
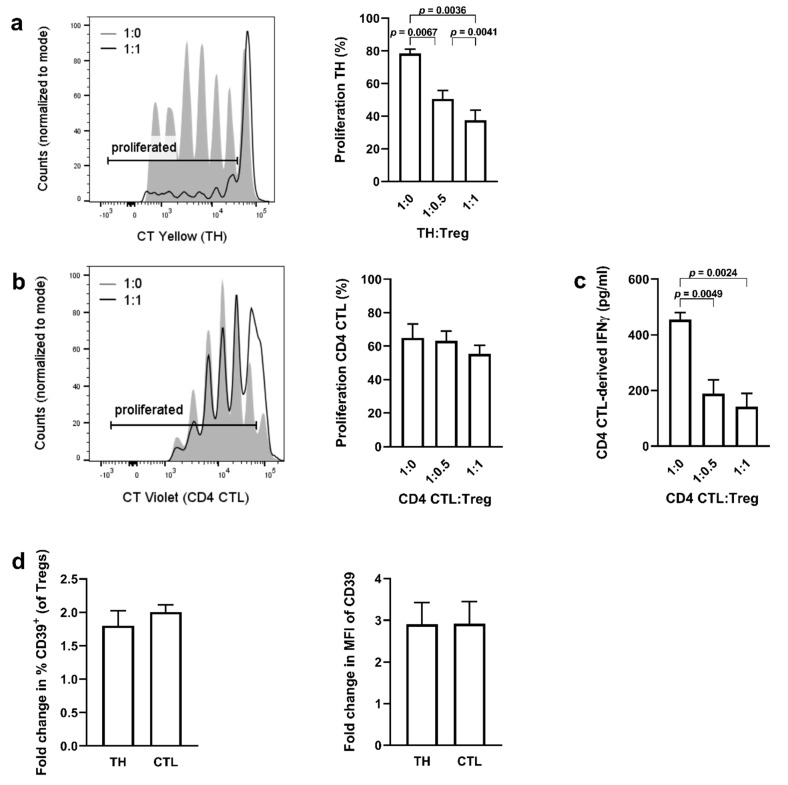
Cytotoxic CD4^+^ T cells are resistant to Treg-mediated suppression of proliferation, but not to Treg-mediated inhibition of IFN-γ production. (**a**–**c**) Representative flow cytometry plot and cumulative data of TH cell proliferation (**a**) when co-cultured with Tregs. Representative flow cytometry plot and cumulative data of CD4 CTL cell proliferation (**b**) and production of IFN-γ (**c**) when co-cultured with Tregs. Error bars indicate mean ± SEM for *n* = 7 donors from seven independent experiments; data analyzed by repeated measures one-way ANOVA, post hoc Tukey. (**d**) Fold change in % of CD39-expressing Tregs and median fluorescent intensity (MFI) of CD39 on Tregs when co-cultured with naïve TH or CD4 CTL. Data are calculated as fold change of CD39 expression in Tregs cultured alone. Error bars indicate mean ± SEM for *n* = 7 donors from seven independent experiments; data analyzed by paired *t*-test.

**Figure 3:**
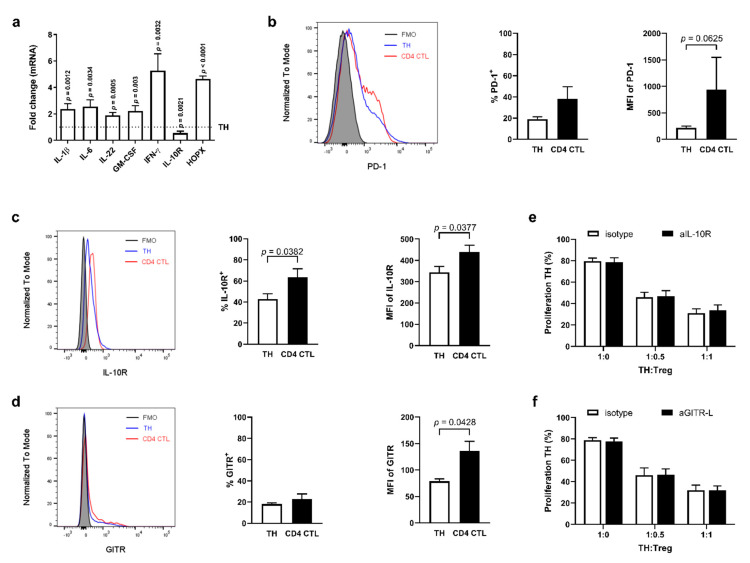
Resistance of Treg-mediated suppression by proinflammatory cytotoxic CD4 T cells is not explained by the effects of IL-10R or GITR. (**a**) Gene expression of *IL-1β*, *IL-6*, *IL-22*, *GM-CSF*, *IFN-γ*, and *HOPX* in stimulated CD4 CTL compared to stimulated TH cells. Error bars indicate mean ± SEM for *n* = 5–8 donors from five independent experiments; data analyzed using mixed model with treatment as fixed effect and sample ID as random effect, post hoc Tukey’s. (**b**–**d**) Representative flow cytometry plots and cumulative data of PD-1 (**b**), IL-10R (**c**), and GITR (**d**) ex vivo expression and median fluorescence intensity (MFI) on TH and CD4 CTL. Fluorescence Minus One (FMO) controls were gated within the TH population. Error bars indicate mean ± SEM for *n* = 5 donors from four independent experiments; data analyzed by Wilcoxon (MFI of PD-1) or paired *t*-test (others). (**e**) Proliferation of TH cells when co-cultured with Tregs in presence of neutralizing antibodies directed against IL-10R (aIL-10R), or a relevant isotype control. Error bars indicate mean ± SEM for *n* = 7 donors from seven independent experiments; data analyzed by two-way ANOVA, post hoc Bonferroni. (**f**) Proliferation of TH cells when co-cultured with Tregs in presence of neutralizing antibodies directed against GITR-ligand (aGITR-L), or a relevant isotype control. Error bars indicate mean ± SEM for *n* = 8 donors from eight independent experiments; data analyzed by two-way ANOVA, post hoc Bonferroni.

To study the interaction between Tregs and naïve TH cells versus CD4 CTL, standard Treg suppression assays were performed with TH cells or CD4 CTL as responders. Details of sorting and gating strategies are given in Figure 1. As expected, Tregs suppressed the proliferation of naïve TH cells (Figure 2a). Tregs were unable to suppress the proliferation of CD4 CTL, but the IFN-γ production of CD4 CTL was decreased by over 50% in the presence of Tregs (Figure 2b,c). To determine the cell−cell contact-dependent suppressive activity of Tregs in the co-cultures, CD39 expression was used as additional readout as it has been shown before that degradation of ATP into adenosine by the CD39/CD73 pathway attributes to the suppressive activity of Tregs [30,42]. We found that the percentage of CD39 expressing Tregs, as well as the median fluorescence intensity (MFI) on Tregs increased to a similar extent when co-cultured with either naïve TH or CD4 CTL (Figure 2d). To investigate the Treg-resistant phenotype of CD4 CTL in further detail, we compared expression profiles of proteins relevant to the evasion of Treg-mediated suppression from CD28^+^ TH cells and CD4 CTL. Upon in vitro stimulation, CD4 CTL displayed a significantly higher mRNA expression of the proinflammatory cytokines interleukin (*IL)-1β*, *IL-6*, *IL-22*, *IFN-γ*, and granulocyte macrophage colony stimulating factor (*GM-CSF*), and prosurvival transcription factor Homeobox-only protein (*HOPX*), while gene expression of the IL-10 receptor (*IL-10R*) was decreased (Figure 3a).

Next, the ex vivo protein expression of the surface molecules IL-10R, glucocorticoid-induced TNFR-related protein (GITR) and programmed cell death protein 1 (PD-1) was analyzed on CD4 CTL and CD28^+^ TH cells. At protein level, the expression of IL-10R was increased on CD4 CTL (Figure 3c). Frequency of GITR-expressing cells did not differ between subsets, although the MFI of GITR was increased on CD4 CTL (Figure 3d). The MFI of PD-1 tended to be increased on CD4 CTL, although the difference in the amount of cells expressing PD-1 did not reach significance (Figure 3b). To further investigate whether IL-10R and GITR expression play a role in resistance to suppression by Tregs in our in vitro suppression assay, co-cultures were performed with naïve TH Tresp with the addition of neutralizing antibodies directed against IL-10R or GITR-ligand (GITR-L; expressed on feeder cells present in the co-culture assay). Blocking IL-10R on naïve TH or blocking GITR-L on feeders did not result in decreased suppression by Tregs (Figure 3e,f), making it unlikely that these two molecules are involved in the resistance of CD4 CTL to Tregs.

### 2.2. Conditioned Medium of CD4 CTL Enhances the Suppressive Phenotype of Tregs, but Decreases Functional Suppression of Naïve TH Cells

**Figure 4:**
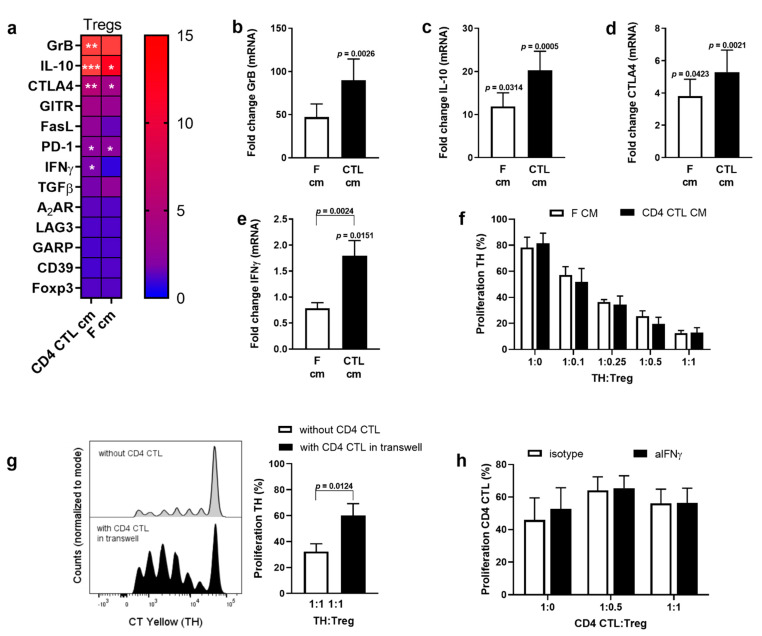
The suppressive phenotype of Tregs is altered upon exposure to the secretome of CD4 CTL. (**a**–**e**) Gene expression of 12 suppression-related genes in sorted Tregs measured after exposure to conditioned medium (CM) of CD4 CTL or feeders. Gene expression is normalized against *CYCA* and *RPL13a*, and calculated as fold change of gene expression in Tregs measured directly upon sorting. Details of gene expression of *granzyme B* (**b**), *IL-10* (**c**), *CTLA-4* (**d**), and *IFN-γ* (**e**) are given. Error bars indicate mean ± SEM for *n* = 10 donors from six independent experiments; data analyzed using mixed model with treatment as fixed effect and sample ID as random effect, post hoc Tukey’s. Exact *p*-values of (**a**) are given in (**b**–**e**) except for PD-1: CD4 CTL cm versus ex vivo *p* = 0.0114, F cm versus ex vivo *p* = 0.0169. (**f**) Proliferation of naïve TH cells when co-cultured with Tregs exposed to CM of CD4 CTL or feeders prior to the suppression assay. Error bars indicate mean ± SEM for *n* = 5 donors from two independent experiments; data analyzed with two-way ANOVA, post hoc Bonferroni. (**g**) Proliferation of naïve TH cells when co-cultured with Tregs, without CD4 CTL, or when co-cultured in a transwell assay with TH cells and Tregs placed in the bottom well and CD4 CTL added to the upper well. See Figure 5a (right panel) for a schematic depiction of the transwell assay setup. Representative flow cytometry plot and cumulative data. Error bars indicate mean ± SEM for *n* = 3 donors from two independent experiments; data analyzed with paired *t*-test. (**h**) Proliferation of CD4 CTL when co-cultured with Tregs and with or without neutralization of Treg-derived IFN-γ. Error bars indicate mean ± SEM for *n* = 5 donors from five independent experiments; data analyzed with two-way ANOVA, post hoc Bonferroni.

**Figure 5:**
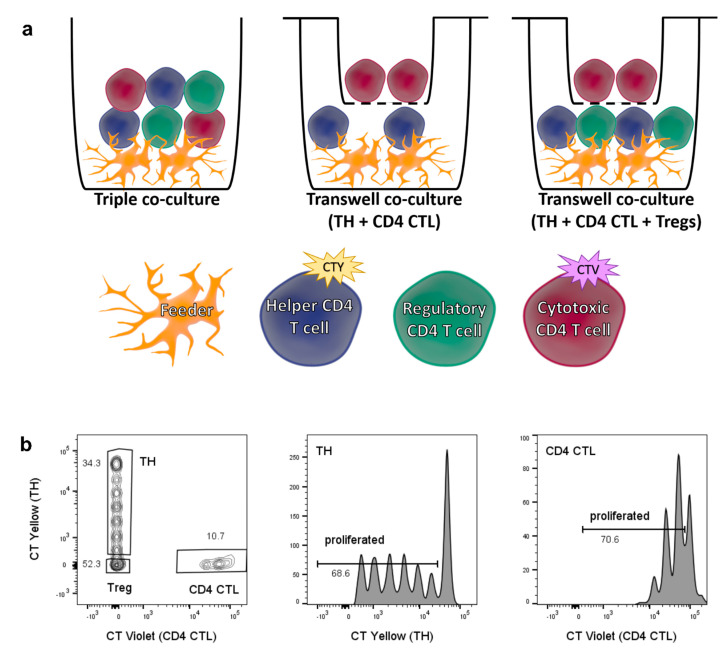
Setup and readout of triple co-culture assay with and without transwell system. (**a**) Schematic depiction of triple co-culture assay (left), transwell assay with naïve TH placed in bottom well and CD4 CTL in upper well (middle), and transwell assay with naïve TH and Tregs placed in bottom well, and CD4 CTL in upper well (right). (**b**) Gating strategy for simultaneous analysis of TH and CD4 CTL cell proliferation in triple co-culture. Single, live CD4^+^ cells are divided into naïve TH cells labelled with CellTrace Yellow (CTY), CD4 CTL labelled with CellTrace Violet (CTV), and unlabeled Tregs.

Next, we investigated the effect of CD4 CTL on Tregs in more detail. When Tregs were activated in the presence of the conditioned medium (CM) of CD4 CTL or feeders, mRNA expression of Granzyme B, IL-10, PD-1, and CTLA-4 increased (Figure 4a–d). Gene expression of IFN-γ in Tregs was induced by CM of CD4 CTL, but not by CM of feeder cells (Figure 4e). Gene expression of other functional molecules thought to be involved in Treg-mediated suppression (Foxp3, GITR, FasL, TGFβ, A2AR, LAG3, GARP, CD39) did not change significantly compared to direct ex vivo levels. To investigate whether the observed changes in gene expression of regulatory markers affected Treg functionality, a co-culture was performed of naïve TH Tresp with Tregs pre-exposed to CM of CD4 CTL. There was no difference in the suppressive capacity of Tregs treated with CM of CD4 CTL or with CM of feeders (Figure 4f). Next, we expanded the suppression assay with a transwell system to study the effect of the CD4 CTL secretome on the suppressive capacity of Tregs towards naïve TH Tresp more directly. The setup of the transwell assay is depicted in Figure 5a. The presence of CD4 CTL in the upper well (no cellular contact) reduced Treg-mediated suppression of naïve TH cells in the bottom well (Figure 4g). Finally, to investigate whether the increase in IFN-γ mRNA in Tregs explained their lack of suppression of cytotoxic CD4 T cells [31,32], Tregs were incubated with neutralizing antibodies directed against IFN-γ prior to co-culture with CD4 CTL. Neutralizing Treg-derived IFN-γ did not reinstall Treg-mediated suppression of CD4 CTL (Figure 4h), while ELISA data of the co-culture supernatant confirmed that all secreted IFN-γ was neutralized (<4 pg/mL).

### 2.3. Conditioned Medium of CD4 CTL Promotes IL-17 Production

**Figure 6:**
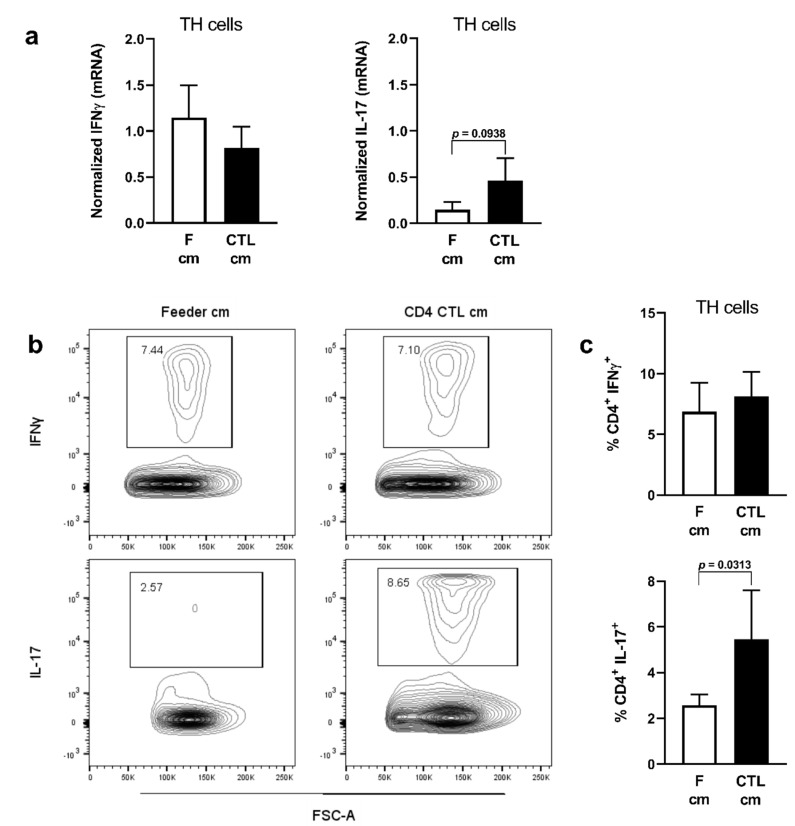
Conditioned medium of CD4 CTL promotes IL-17 production in memory TH cells. (**a**–**c**) Gene (**a**) and protein (**b**), representative flow cytometry plot, and (**c**) cumulative data expression of IFN-γ and IL-17 measured in memory TH cells after stimulation with conditioned medium (cm) of CD4 CTL or feeders. Gene expression data is normalized against *RPL13a*. Error bars indicate mean ± SEM for *n* = 6 donors from four independent experiments; data analyzed with Wilcoxon test.

As we found that Treg-resistant CD4 CTL express a range of proinflammatory genes, they may also directly affect the polarization of CD28^+^ TH cells. To investigate this, we exposed memory TH cells to CD4 CTL CM and assessed the TH phenotype of these cells based on IFN-γ (TH1) and IL-17 (TH17) expression. At gene expression level, there was a trend towards induction of *IL-17* expression, while *IFN-γ* expression was not affected (Figure 6a). Flow cytometric analyses revealed that CM of CD4 CTL modestly but significantly increased IL-17 production by memory TH cells, while IFN-γ production remained unchanged (Figure 6b,c).

### 2.4. Cell−Cell Interaction between CD28^+^ TH Cells and CD4 CTL Reverts the Proliferation-Inducing Effects of the CD4 CTL Secretome

To better understand the interaction between CD4 CTL, CD28^+^ TH cells and Tregs, co-culture assays were performed with these three cell types simultaneously. Additionally, CD4 CTL and naïve TH cells were co-cultured in a transwell system. The experimental setup is depicted in Figure 5a (left and middle panel). This allowed us to analyze CD4 CTL-mediated effects facilitated by cell−cell contact. For the triple co-culture setup, sorted naïve TH cells labeled with CellTrace Yellow, CD4 CTL labeled with CellTrace Violet, and unlabeled Tregs were cultured together and analyzed after five days, with the different labeling dyes permitting for gating of all three subsets (Figure 5b).

**Figure 7:**
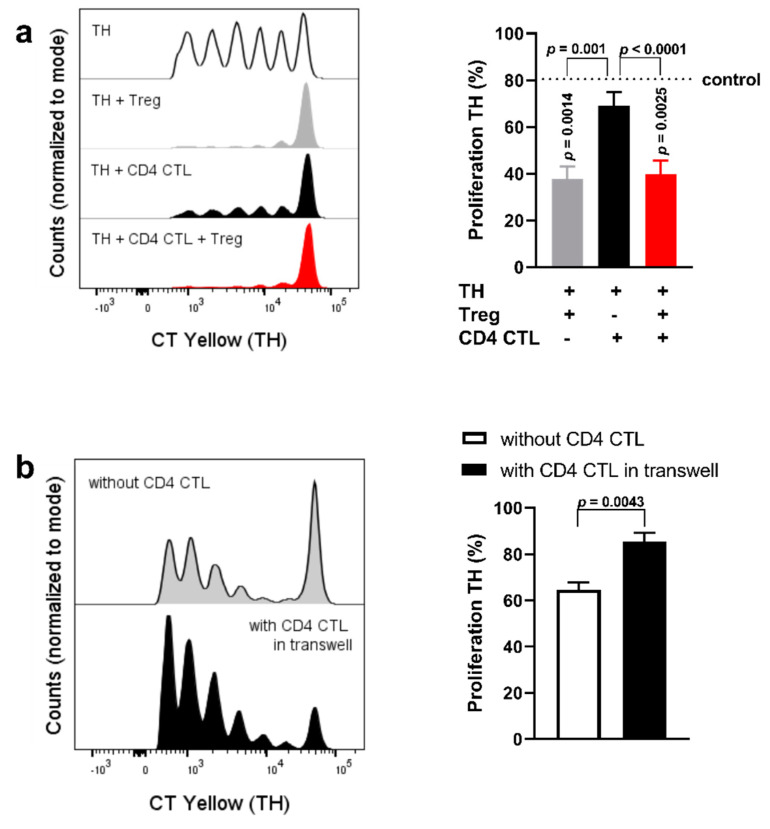
Cell−cell interaction between naïve TH cells and CD4 CTL prevents the proliferation-inducing effects of the CD4 CTL secretome. (**a**) Representative plot and cumulative data of proliferation of naïve TH cells when co-cultured with Tregs, or with CD4 CTL, or with Tregs and CD4 CTL. The control (dotted line) indicates the proliferation of naïve TH cells when cultured without Tregs and CD4 CTL, which is also displayed in the uppermost panel of the representative plot. Error bars indicate mean ± SEM for *n* = 7 donors from seven independent experiments; data analysed with repeated measures one-way ANOVA, post hoc Tukey. (**b**) Proliferation of naïve TH cells when cultured alone or co-cultured in a transwell assay with TH cells placed in the bottom well and CD4 CTL in the upper well. Representative flow cytometry plot and cumulative data. Error bars indicate mean ± SEM for *n* = 3 donors from two independent experiments; data analyzed with paired *t*-test.

Given that the CD4 CTL secretome reduced the suppression of naïve TH (Figure 4g), we focused on the proliferation of naïve TH cells in this triple co-culture setup. When co-cultured with CD4 CTL, the proliferation of naïve TH cells remained comparable to the proliferation rates of naïve TH cells cultured alone. Furthermore, proliferation of naïve TH cells was suppressed in the triple co-culture system to rates comparable to TH cell proliferation when cultured with Tregs only (Figure 7a). Interestingly, when naïve TH cells were exposed to the secretome of CD4 CTL without cell-to-cell contact, proliferation of these TH cells was increased (Figure 7b). Allowing for cell−cell interaction between CD4 CTL and naïve TH, therefore, appeared to be sufficient to prevent the proliferation-inducing effects of the CD4 CTL secretome.

## 3. Discussion

In this study, we sought for explanations for the previously reported resistance of CD4 CTL against Treg-mediated suppression [6]. First, we confirmed the reported differences in susceptibility to Treg-mediated suppression between TH and CD4 CTL [6]. To find leads that would explain this, we compared the phenotype of CD4 CTL and conventional CD28^+^ TH cells. Unexpectedly, CD4 CTL showed an upregulation of IL-10R protein, while mRNA expression levels were decreased. Additionally, an upregulation of HOPX and PD-1 was found. We further investigated the role of IL-10R in our in vitro system and found that blocking IL-10R did not affect Treg-mediated suppression. This is in accordance with other studies in which neutralization of IL-10 did not abrogate suppression in vitro [19,43,44,45]. Blocking of GITR ligation did not affect suppression of conventional TH cells. This is in accordance with Nocentini et al., who claim that GITR-mediated suppression is only present in mice and not in humans [40]. The upregulation of HOPX reported here could indicate that CD4 CTL are resistant to apoptosis. HOPX is described to convey resistance to apoptosis via the evasion of Fas-mediated apoptosis and, respectively, up- and downregulation of anti- and proapoptotic molecules [39]. Studies have shown that Tregs can utilize Fas/Fas-ligand signaling to suppress proliferation of CD8^+^ effector T cells [26,46], but we did not find an increased expression of Fas-ligand on Tregs. CD4 CTL are known to express the anti-apoptotic molecules BCL-2 and cFLIP [47], which is in accordance with an increased expression of HOPX. Expression of the co-inhibitory molecule PD-1 was increased in CD4 CTL when analyzing MFI, and increased PD-1 expression is considered a hallmark of exhausted T cells [48]. However, we observed proliferation rates in CD4 CTL comparable to those of conventional TH cells, indicating that the CD4 CTL classified by loss of CD28 expression should not be considered exhausted T cells. The absence of CD28 itself can provide another possible explanation why CD4 CTL resist Treg-mediated suppression. CTLA4 on Tregs, which was upregulated after exposure to CM of CD4 CTL, binds to B7 molecules expressed on antigen-presenting cells [41]. Additionally, Treg-derived CTLA4 can induce a downregulation of B7 molecules on antigen-presenting cells [49]. These processes hamper stable interactions between B7 molecules and CD28 on effector T cells, diminishing TH activation and proliferation [16]. Since CD4 CTL do not express CD28, blockade or downregulation of B7 should not affect them. We did not investigate this interaction here, but inducing re-expression of CD28 on CD4 CTL, or blocking of CD28 on conventional TH cells could answer this question in the future. Based on our findings, we propose that the reduced need for CD28-mediated co-stimulation and upregulated expression of the anti-apoptotic molecule HOPX provide possible explanations for the observed reduced susceptibility of CD4 CTL to Treg-mediated suppression in vitro.

Next to increased survival mechanisms, we expected that CD4 CTL would exhibit a more proinflammatory phenotype than their conventional TH counterparts. CD4 CTL are defined as TH1-like cells, since they produce high levels of IFN-γ and TNFα, but little or no IL-4 or IL-17 [50,51]. We can confirm here that activated CD4 CTL have a high expression of IFN-γ, and show that they also express IL-22 and GM-CSF, which are usually produced by TH17 cells. In line with our findings, it has been shown that TH1 and CD4 CTL cells can produce these cytokines in the absence of IL-17 production [10,52,53]. Additionally we showed that conditioned medium of CD4 CTL induces a slight increase in IL-17 production, likely due to IL-1β, IL-6, and GM-CSF [37,54,55]. This was however not accompanied by an increase in the TH17-regulating transcription factor RORγt (data not shown). Interestingly, the secretome of CD4 CTL promoted proliferation of conventional TH cells, but this effect was abolished by cell−cell contact between CD4 CTL and conventional TH cells. The increased proliferation of conventional TH cells might be induced by the high levels of IFN-γ secreted by CD4 CTL, either via enhancing TH cell survival [56] or via enhancing stimulation provided by feeder cells in our assay [57]. The observed effects of cell−cell contact between CD4 CTL and conventional TH cells might indicate that CD4 CTL express inhibitory surface molecules that prevent excessive proliferation of conventional TH cells. An obvious candidate is the IL-2 receptor CD25, given its role in Treg-mediated suppression [58,59]. CD4 CTL do not express CD25 ex vivo [47,60], but upregulate CD25 upon stimulation to expression levels comparable to their CD28^+^ counterparts [47]. As it has been reported that CD4 CTL do not produce IL-2 [60], activated CD4 CTL might bind to IL-2 produced by conventional TH cells and thereby deprive these cells of IL-2. However, other studies show that CD4 CTL are not dependent on IL-2 for their survival [47,61]. It should be investigated further if IL-2 deprivation indeed is how CD4 CTL inhibit excessive proliferation of conventional TH cells.

We next investigated the interaction between Tregs and CD4 CTL in more detail. Tregs exposed to CD4 CTL conditioned medium had an increased gene expression of IFN-γ. IFN-γ producing Tregs have a reduced suppressive capacity and blocking this cytokine recovers their suppressive capacity to some extent [31,62]. However, in our hands, blocking Treg-derived IFN-γ did not reinstall suppression of CD4 CTL. Resistance to Treg-mediated suppression has been reported for effector TH cells in autoimmune disease like multiple sclerosis and type 1 diabetes, and was reported to involve granzyme B and IL-6 [33,38,63]. However, neutralizing granzyme B or IL-6 in our co-culture setup did not reinstall Treg-mediated suppression of CD4 CTL (data not shown). While the conditioned medium of CD4 CTL was sufficient to enhance the suppressive phenotype in Tregs at the gene level, exposing Tregs to the CD4 CTL secretome in a transwell setup decreased their suppression of conventional TH cells. Given our other findings, it is likely that secreted factors by CD4 CTL promote proliferation of conventional TH cells, instead of directly diminishing the suppressive capacity of Tregs. Our observation of the Treg-mediated decrease in IFN-γ production of CD4 CTL, and the lack of differences in CD39 expression of Tregs co-cultured with either conventional TH Tresp or CD4 CTL Tresp, underscore that these Tregs remain functional, but somehow fail to stop proliferation of CD4 CTL.

Taken together, we have shown here that CD4 CTL have a proinflammatory phenotype and express molecules indicative of resistance to apoptosis. We demonstrated that the CD4 CTL secretome induced TH17 polarization and proliferation of conventional TH. The proliferation-inducing effects were however abolished by cell−cell contact. Our results suggest that the resistance of CD4 CTL to Treg-mediated suppression lies within the CD4 CTL themselves, rather than being explained by the dysfunction of Tregs, but further studies are necessary to identify relevant pathways. We propose that CD4 CTL promote their own maintenance by becoming resistant to Treg suppression and creating a proinflammatory microenvironment in which proliferation of other TH cells is inhibited in their direct vicinity. This is supported by findings that CD4 CTL accumulate in autoimmune diseases [9,12,64]. Further research is warranted to investigate whether these in vitro findings translate to in vivo memory inflation of CD4 CTL, a phenomenon that has already been described for cytomegalovirus-specific CD8^+^ T cells [65].

## 4. Materials and Methods

Study subjects—Peripheral blood samples were collected from healthy controls in collaboration with the University Biobank Limburg (UBiLim, Hasselt, Belgium). In total, 39 unique donors were used, of which a subset of 11 donors was used for experiments with CD4 CTL. These donors had 2.9–17.2% CD4^+^CD28^–^ (mean: 7.1%) within their total peripheral CD4^+^ T cell population. For the donors without CD4 CTL expansion (n = 28; 8 males and 20 females) ages ranged between 22 from 58 years (mean ± SEM: 31.6 ± 1.7). For the donors with CD4 CTL expansion (n = 11; 8 males and 3 females) ages ranged between 28 and 67 years (mean ± SEM: 45.6 ± 4.3). This study was approved by the local ethical committee and informed consent was obtained from all donors.

Cell isolation and flow cytometry—All experiments were done using fresh PBMC isolated from whole blood by density gradient centrifugation (Cedarlane lympholyte, Sheffield, UK). Unless indicated otherwise, positive selection of CD4^+^ T cells from PBMCs using magnetic beads was performed prior to sorting according to the manufacturer’s protocol (MACS CD4 microbeads, Miltenyi Biotec, Bergisch Gladbach, Germany). The untouched CD4^–^ portion of PBMCs was irradiated to use as feeders. Cells were sorted using a FACSAriaII, cell phenotyping data was acquired using a BD LSRFortessa, and all flow cytometry data were analyzed using FlowJo 10.6.2 (all from BD Biosciences, Erembodegem, Belgium). For sorting of CD4 CTL (CD4^+^CD28^–^), total TH (CD4^+^CD28^+^), naïve TH (CD4^+^CD28^+^CD45RO^–^), and Tregs (CD4^+^CD28^+^CD25^hi^CD127^low^) and for phenotyping cells, the following antibodies were used: CD4 PE-Cy7, CD4 APC eFluor780, CD28 APC, CD127 PE, PD-1 PE-Cy7, IL-17a PE (all Thermo Fisher Scientific, Waltham, MA, USA), CD25 PE Cy7, CD28 BV711, CD39 BV711, GITR PE, IL-10R BV421 (all BioLegend, San Diego, CA, USA), CD28 PE, IFN-γ PE-CF594 (both BD Biosciences, Erembodegem, Belgium). The sorting strategy is depicted in Figure 1, and purity of all sorts was confirmed to be >95%.

Cell culture and stimulation assays—Cells were cultured at 37 °C/5% CO_2_ in RPMI-1640 medium (Lonza, Basel, Switzerland) supplemented with 10% fetal bovine serum (FBS; Gibco, Thermo Fisher Scientific, Waltham, MA, USA), 1% nonessential amino acids, 1% sodium pyruvate, 50 U/mL penicillin, and 50 mg/mL streptomycin (all Life Technologies, Merelbeke, Belgium).

Sorted CD4 CTL or sorted CD4^+^CD28^+^ TH were stimulated in U-bottomed 96-well plates with 2 µg/mL anti-CD3 (clone OKT3, Thermo Fisher Scientific, Waltham, MA, USA) and 0.1 U/mL IL-2 (Roche Diagnostics, Basel, Switzerland) at a density of 1 × 10^5^ cells, in the presence of irradiated autologous feeders (1:1). As a control, only feeders were plated and stimulated. After five days supernatant was collected and stored at −80 °C (hereafter referred to as conditioned medium (CM)), and cells were pelleted for mRNA analyses.

Sorted Tregs were either pelleted for mRNA analyses directly ex vivo or stimulated in U-bottomed 96-well plates with 1 µg/mL anti-CD3 (clone OKT3, Thermo Fisher Scientific, Waltham, MA, USA), 1 µg/mL anti-CD28 (clone CD28.2, BD Biosciences, Erembodegem, Belgium) and 25 U/mL IL-2 (Roche Diagnostics, Basel, Switzerland) at a density of 1 × 10^5^ cells, with or without 50% (*v*/*v*) CM of stimulated CD4 CTL or feeders. After 72 h, supernatant was removed and cells were pelleted for mRNA analyses.

Memory CD4^+^ T cells were magnetically isolated from human PBMCs according to the manufacturer’s protocol (MagniSort Human CD4 memory T cell Enrichment Kit, Thermo Fisher Scientific, Waltham, MA, USA). Cells were plated in 24-wells plates at 5 × 10^5^ cells/well, stimulated with plate-bound 2.5 µg/mL anti-CD3 (clone OKT3, Thermo Fisher Scientific, Waltham, MA, USA) and soluble 2 µg/mL anti-CD28 (clone CD28.2, BD Biosciences, Erembodegem, Belgium), supplemented with 50% (*v*/*v*) CM of CD4 CTL or feeders. After five days, supernatant was removed and cells were pelleted for mRNA analyses. Cells for flow cytometric analyses were restimulated for 4 h with 1 µg/mL calcium ionomycin (Sigma-Aldrich, Saint Louis, MO, USA), 1 µL/mL Golgiplug (BD Biosciences, Erembodegem, Belgium), and 25 ng/mL PMA (Sigma-Aldrich, Saint Louis, MO, USA) in fresh culture medium.

Treg suppression assay—Sorted T responder cells, defined as CD4 CTL Tresp or naïve TH Tresp, were labelled with 5 μM CellTrace Violet or CellTrace Yellow respectively (both Thermo Fisher Scientific, Waltham, MA, USA) prior to culturing the cells alone (2 × 10^4^ cells/well; referred to as 1:0), or together, and/or with Tregs. Ratios at which the different cell types were combined are specified in the appropriate figure legends; triple co-cultures consisted of autologous CD4 CTL, naïve TH, and Tregs. Gating strategy for triple co-cultures is depicted in Figure 5b. Proliferation controls exist of Tresp cultured alone in a cell density comparable with the highest cell density of other conditions in the same experiment. Cells were stimulated with 2 µg/mL anti-CD3 (clone OKT3, Thermo Fisher Scientific, Waltham, MA, USA), 2 U/mL IL-2 (Roche Diagnostics, Basel, Switzerland), and irradiated autologous feeders (1 × 10^5^ cells) for 5 days, after which supernatant was collected and stored at −80 °C, and CellTrace dilution was determined using flow cytometry. The 96-wells plates equipped with 0.4 um pore size transwell (Millicell-96 Cell Culture Insert Plate; Merck, Darmstadt, Germany) were used to perform suppression assays in the transwell system. For co-cultures where neutralizing or suppressive agents were tested, the culture medium was supplemented with the following compounds or appropriate controls: 10 µg/mL anti-human-IFN-γ (R&D systems, Minneapolis, MN, USA) or mouse anti-human IgG2a (BioLegend, San Diego, CA, USA), 7 µg/mL anti-GITRL (R&D systems, Minneapolis, MN, USA) or goat anti-human IgG (Jackson ImmunoResearch Europe Ltd., Cambridgeshire, UK), 40 µg/mL anti-IL-10R (BioLegend, San Diego, CA, USA) or rat anti-human IgG2a (BioLegend, San Diego, CA, USA). With the exception of anti-IFN-γ, these compounds were added directly to the co-culture. For anti-IFN-γ, Tregs were pre-incubated with the neutralizing antibody for 1 h, after which 2 µg/mL anti-CD3 (clone OKT3, Thermo Fisher Scientific, Waltham, MA, USA), 2 U/mL IL-2 (Roche Diagnostics, Basel, Switzerland), feeders and Tresp were added directly to the treated Tregs. For experiments in which Tregs were exposed to CM prior to the start of the co-culture, sorted Tregs were incubated with 50% (*v*/*v*) CM of CD4 CTL or feeders for 24 h, in the presence of 2 µg/mL anti-CD3 (clone OKT3, Thermo Fisher Scientific, Waltham, MA, USA), 2 U/mL IL-2 (Roche Diagnostics, Basel, Switzerland), and feeders. After 24 h the supernatant was removed, naïve TH Tresp were added to the Tregs in fresh culture medium containing 2 µg/mL anti-CD3 (clone OKT3, Thermo Fisher Scientific, Waltham, MA, USA) and 2 U/mL IL-2 (Roche Diagnostics, Basel, Switzerland), and cells were cultured for 5 days. Where specified, IFN-γ was determined in the co-culture supernatant using the IFN-γ Ready-set-go ELISA kit according to the manufacturers’ protocol (Thermo Fisher Scientific, Waltham, MA, USA).

qPCR—Pelleted cells were washed once with 1 × PBS (Lonza, Basel, Switzerland) and stored at −80 °C until mRNA isolation. mRNA was prepared using the RNeasy mini kit (Qiagen, Hilden, Germany), according to manufacturer’s instructions. RNA concentration and quality was determined with a NanoDrop spectrophotometer (Isogen Life Science, IJsselstein, The Netherlands). RNA was reverse transcribed to cDNA using the qScriptTM cDNA synthesis kit (Quanta Biosciences, Gaithersburg, MD, USA). Quantitative PCR was subsequently conducted on a StepOnePlus detection system (Applied biosystems, Gaasbeek, Belgium). Relative quantification of gene expression was performed using the comparative Ct method. Data were normalized to the most stable reference genes (*CYCA* and *RPL13a*). Primers were chosen according to literature or designed using Primer-Express (http://www.ncbi.nlm.nih.gov/tools/primer-blast, accessed on 19 February 2019), and details of primers used are shown in Table 1.

Statistical analysis—Statistical analyses were performed using GraphPad Prism version 8.4.1 (GraphPad Software, San Diego, CA, USA) and SAS JMP Pro 13 (SAS Institute, Cary, NC, USA). Prior to statistical analysis, the Gaussian distribution of datasets was tested using Shapiro−Wilk test. Dixon’s test with α = 0.05 was used to detect extreme values. Details of statistical tests are given in figure legends. Cumulative data are shown as mean ± SEM. A *p*-value < 0.05 was considered significant.

## Figures and Tables

**Table 1:** Forward and reverse primer sequence of human qPCR primers.

Gene	Full Name	Forward Primer	Reverse Primer
*A2AR*	Adenosine A2A receptor	CGCTCCGGTACAATGGCTT	TTGTTCCAACCTAGCATGGGA
*CD39*	Ectonucleoside triphosphate diphosphohydrolase-1	ACTATCGAGTCCCCAGATAATGC	CCTGATCCTTCCCATAGCACAA
*CTLA4*	Cytotoxic T-lymphocyte-associated protein 4	TGGATTTCAGCGGCACAAGGCT	CTGGGCCACGTGCATTGCTTTG
*CYCA*	Cyclin A	AGACTGAGTGGTTGGATGGC	TCGAGTTGTCCACAGTCAGC
*FasL*	Fas ligand	AAAGTGGCCCATTTAACAGGC	AAAGCAGGACAATTCCATAGGTG
*Foxp3*	Forkhead box P3	GCACCAGCTCTCAACGGTGGATG	GAAGACCCCAGTGGCGGTGG
*GARP*	Glycoprotein A Repetitions Predominant	AGACCCTTGATCTATCTGGGAAC	GAAGCTGATCTCATTGGTGCT
*GITR*	glucocorticoid-induced TNFR-related protein	CGAGTGGGACTGCATGTGTG	GGCAGGTCGTGCAGCAA
*GM-CSF*	Granulocyte-macrophage colony-stimulating factor	CCAGGAGCCGACCTGCCTACA	GAAGTTTCCGGGGTTGGAGGGC
*GrB*	Granzyme B	GCGAATCTGACTTACGCCATTA	CCAGAGTCCCCCTTAAAGGAA
*HOPX*	Homeodomain-only protein	TCAACAAGGTCGACAAGCAC	TCTGTGACGGATCTGCACTC
*IFN-γ*	Interferon-γ	GGGGCCAACTAGGCAGCCAAC	AAGCACTGGCTCAGATTGCAGGC
*IL-1β*	Interleukin-1β	GATGAAGTGCTCCTTCCAGG	GCATCTTCCTCAGCTTGTCC
*IL-6*	Interleukin-6	GAGGAGACTTGCCTGGTGAA	GCTCTGGCTTGTTCCTCACT
*IL-10*	Interleukin-10	GCTGTCATCGATTTCTTCCC	ATAGAGTCGCCACCCTGATG
*IL-10Rα*	Interleukin-10Rα	CCTCCGTCTGTGTGGTTTGAA	CACTGCGGTAAGGTCATAGGA
*IL-17*	Interleukin-17	ATGGCCCAGCCATGGTCAAGTA	GCACAGGCGGGCAACTCTCA
*IL-22*	Interleukin-22	AACCGCACCTTCATGCTGGCT	CGCTCACTCATACTGACTCCGTGG
*LAG3*	Lymphocyte-activation gene 3	GCCTCCGACTGGGTCATTTT	CTTTCCGCTAAGTGGTGATGG
*PD-1*	Programmed cell death protein 1	CTCAGGGTGACAGAGAGAAG	GACACCAACCACCAGGGTTT
*RPL13A*	Ribosomal Protein L13a	AAGTTGAAGTACCTGGCTTTCC	GCCGTCAAACACCTTGAGAC
*TGFβ*	Transforming growth factor β	GTGGAAACCCACAACGAAAT	CACGTGCTGCTCCACTTTTA

## Data Availability

Data generated in this study can be obtained by contacting the corresponding author.
